# A pilot study of intraoperative melphalan to prevent recurrent PVR: the IOMPVR study

**DOI:** 10.1186/s40942-025-00675-4

**Published:** 2025-05-01

**Authors:** Carlos A. Moreira-Neto, Jose S Pulido, Luca Bongiovanni de Miranda Gonçalves, Gabriel Nunes Cavalcanti, Acacio Souza Lima, José de Paula Barbosa Neto, Daniel Lavinsky, Talita Virginia Fernandes de Oliveira, Fernando Ejii Ogata, Lucas Zago Ribeiro, Luiz Fernando Teixeira, Flavia Borelli Gomes do Nascimento, Luiz H. Lima, Octaviano Jr. Magalhães, Michel Eid Farah, Maurício Maia

**Affiliations:** 1 Department of Ophthalmology, Vitreoretinal Unit, Federal University of São Paulo, UNIFESP/EPM), 821, Botucatu Sreet, Vila Clementino, São Paulo, Brazil04023-062 Brazil; 2 Vitreoretinal Unit, Hospital de Olhos do Parana, Parana, Brazil; 3 Department of Ophthalmology, Vitreoretinal Unit, Wills Eye Institute, Philadelphia, PA USA; 4 Vision Institute, Sao Paulo, Brazil; 5 Department of Ophthalmology, Ocular Pharmacology Unit, Federal University of São Paulo, UNIFESP/EPM), São Paulo, Brazil; 6 Eyepharma, Pharmaceutical Industrry, Sao Paulo, Brazil; 7 Department of Ophthalmology, Ocular Oncology Unit, Federal University of São Paulo, UNIFESP/EPM), São Paulo, Brazil; 8 Grupo apoio ao Adolescente e Crianca com Cancer (GRAAC), São Paulo, Brazil; 9 Brazilian Institute of Fight Against Blindness (INBRACE)– Assis and Presidente Prudente, Sao Paulo, Brazil

**Keywords:** Melphalan, Pars plana vitrectomy, Proliferative vitreoretinopathy, R hegmatogenous retinal detachment

## Abstract

**Background:**

Proliferative vitreoretinopathy (PVR) is a major cause of failure in cases of retinal detachment (RD) repair. Intravitreal melphalan, a known inhibitor of cellular proliferation, offers a novel therapeutic approach to reduce PVR recurrence and improve outcomes. We evaluated the safety and efficacy of 5 µg/0.1 ml intravitreal melphalan at the end of pars plana vitrectomy (PPV) before silicone oil (SO) injection in eyes with primary PVR related to rhegmatogenous retinal detachments (RRDs) with a minimal 90-day follow-up period.

**Methods:**

This prospective, cross-sectional, interventional pilot study was conducted at the Department of Ophthalmology of the Federal University of São Paulo in patients with primary RRD and PVR. Patients were included who were aged 18 to 85 years with PVR grade CP2 or worse secondary to RRDs in eyes without having undergone a previous RRD surgery. They underwent PPV + scleral buckle + fluid air exchange followed by intravitreal injection of 5 µg/0.1 ml melphalan (270 mOsm) and SO injection.

**Results:**

Six eyes of six patients were enrolled. Ocular examination and imaging showed no retinal toxicity. The logarithm of the minimum angle of resolution best-corrected visual acuity improved from the mean ± standard deviation preoperatively of 2.11 ± 0.22 to 0.89 ± 0.37 at 30 and to 0.84 ± 0.42 at 90 days postoperative (*P* < 0.001). Optical coherence tomography identified intraretinal cysts in five of six eyes and outer retinal layer loss in all study eyes. Only one of six eyes developed a recurrent localized RD on day 90 unrelated to recurrent PVR. PVR recurrence was not observed during the study follow-up.

**Conclusions:**

In this pilot study, the preliminary data showed that PPV followed by intravitreal injection of 5 µg/0.1 ml melphalan was not related to ocular toxicity. The absence of PVR recurrence at 3 months follow-up in these complex PVR eyes is an interesting finding that justifies further investigation.

**Supplementary Information:**

The online version contains supplementary material available at 10.1186/s40942-025-00675-4.

## Introduction

Proliferative vitreoretinopathy (PVR) is the most common cause of failure in rhegmatogenous retinal detachment (RRD) repair [1, 2, 3, 4, 5]. The incidence rates of PVR following a primary RRD surgery range from 5.1–11.7% [6–8]. Currently, the surgical options for RRD and PVR are pneumatic retinopexy, scleral buckle, and pars plana vitrectomy (PPV) [9]. PPV is considered the standard treatment for PVR, as recurrent vitreoretinal traction can lead to retinal re-detachment, significant visual loss, and phthisis bulbi [9, 10].

The pathophysiology of PVR is characterized by blood-retinal barrier breakdown and cellular proliferation of retinal pigment epithelial (RPE) cells, astrocytes, fibroblasts, myofibroblasts, and macrophages [9, 10, 11, 12, 13]. Despite advances in surgical techniques, a significant number of recurrent retinal detachments (RDs) result from PVR, requiring research into other therapeutic options that act on the disease pathophysiology by inhibiting cellular proliferation and membrane contraction [9, 10, 11, 12, 13, 14].

Melphalan (L-phenylalanine mustard or L-PAM), an anticancer agent introduced in 1957, is a nitrogen mustard alkylating agent that induces cellular damage by alkylation of DNA bases, resulting in DNA molecule breakage and cross-linking. Melphalan blocks tumor cell growth by inhibiting nucleic acid biosynthesis and bone marrow suppression with a dose-dependent effect [15, 16, 17, 18]. The pathophysiology and risk factors for PVR have guided investigations for molecular targets. Drugs that counteract inflammation, growth factors, and especially cellular proliferation are the leading candidates for treating PVR [9, 10, 14]. Because melphalan is a potent inhibitor of nucleic acid biosynthesis resulting in inhibition of cellular proliferation, there is an important rationale to use this drug for PVR inhibition that was not reported previously in the medical literature (MEDLINE search, July 9, 2024).

Melphalan is also an intraocular chemotherapeutic agent in infants with retinoblastoma [19]. Because of the intraocular safety profile in these infants, we hypothesized that it might be a therapeutic option for inhibiting PVR [10, 11, 12, 13]. Therefore, the purpose of this study was two-fold: to assess the safety of intravitreal melphalan injection in the setting of vitreoretinal surgery for RRD with PVR and to evaluate PVR recurrence.

## Methods

This study was approved by the Institutional Review Board (number: 5.282.168) at the Federal University of São Paulo, São Paulo, Brazil. The data were stored and managed in compliance with guidelines from the Brazilian General Data Protection Law and adhered to the tenets of the Declaration of Helsinki.

All procedures were conducted in accordance with ethical standards, including obtaining informed consent and ensuring confidentiality and anonymity.

### Study population

This prospective, cross-sectional, interventional study was conducted at the Department of Ophthalmology of the Federal University of São Paulo in patients with primary RRD and PVR. Patients were informed about their ocular condition and the off-label use of intravitreal injection of 5 µg/0.1 ml of melphalan in cases of RRD with PVR. The research protocol was explained to each patient and those interested were asked to provide informed consent.

### Inclusion/exclusion criteria

Subjects were included if they were 18 to 85 years old and had a primary RRD with a CP2 PVR grade or worse according to the Retina Society Classification of 1991 [20]. Other inclusion criteria included agreeing to and providing informed consent and having normal preoperative results for complete blood count, urea, glucose, creatinine, prothrombin time and activity, coagulation time, and electrocardiography.

Exclusion criteria included media opacification at the screening visit that prevents clinical/photographic evaluation and documentation; any condition or situation that could confound the results or significantly interfere with patient participation; a history of allergy to fluorescein dye or povidone-iodine; lack of cooperation for obtaining the best-corrected visual acuity (BCVA); a previous ocular surgery, except cataract surgery; ocular disease such as diabetic retinopathy, retinal vascular occlusions, and macular degenerations or dystrophies; and pregnancy or breast-feeding.

The BCVA was measured and converted to the logarithm of the minimum angle of resolution (logMAR) acuity using vision correction based on automatic refraction [21, 22, 23]. Anterior segment biomicroscopy and applanation tonometry were performed by calibrated Goldmann tonometer at all visits.

The clinical features were studied using a combination of color fundus photography, fundus autofluorescence (FAF), fluorescein angiography (FA), and spectral-domain optical coherence tomography (SD-OCT) or swept-source OCT (SS-OCT). Imaging was performed using the available technology at the time of the visit. All patients had at least one visit during their disease course when FAF and SD-OCT or SS-OCT imaging were acquired. These tests were conducted to assess the safety of intravitreal melphalan injection during vitreoretinal surgery for RRD with PVR and to evaluate PVR recurrence, retinal reattachment rates, BCVA, and other clinical and complementary examination findings over a 3-month follow-up period.

**Fig. 1:**
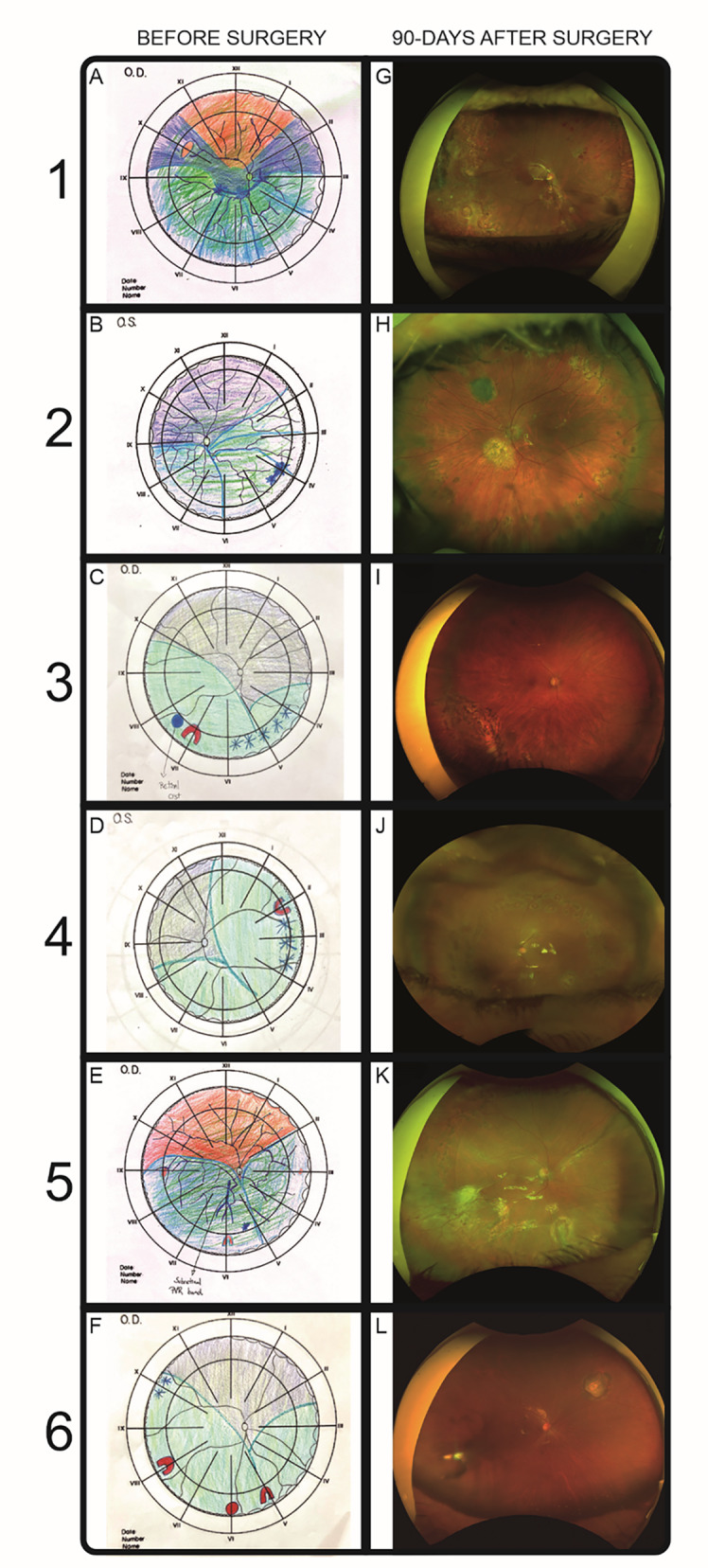
The first column contains preoperative fundus drawings of all six patients. The second column contains UWF color fundus images of all six patients obtained on postoperative day 90. Patient (1) A, The preoperative drawing shows vitreous haze and a macula off and inferior retinal detachment with a star pattern of PVR CP6. G,–A 90-day postoperative UWF color fundus image shows a re-detachment of the inferior retina due to a posterior retinal tear. Laser scars are seen in the periphery. There are no signs of recurrent PVR. Patient (2) B, A preoperative drawing shows vitreous haze and a complete retinal detachment because of a PVR CP3. H,–A 90-day postoperative UWF color fundus image shows a completely reattached retina. Laser scars are seen in the periphery and around the retinotomies. There are no signs of PVR recurrence. Patient (3) C, a preoperative drawing shows vitreous haze, a complete retinal detachment with a tear at the 7 o’clock position, PVR CP3, and a temporal retinal cyst. I, A 90-day postoperative UWF FAF image shows a completely reattached retina. Laser scars are seen in the inferior retina. Patient (4) D, A preoperative drawing shows vitreous haze, a complete retinal detachment with a tear at the 2 o’clock position, and PVR CP2. J, a 90-day postoperative UWF color fundus image shows a completely reattached retina and laser scars in the mid-periphery. Patient (5) E, a preoperative drawing shows vitreous haze, an inferior retinal macula-off detachment because of PVR CP6, and tears at the 3, (6) and 9 o’clock positions. K, A 90-day postoperative UWF color fundus image shows that the retina is reattached with no signs of PVR recurrence. Patient 6. F, a preoperative drawing shows vitreous haze, a complete retinal detachment because of PVR CP2, and tears at the 5, 6, and 8 o’clock positions. L, a 90-day postoperative UWF color fundus image shows a completely reattached retina and laser scars around the retinotomy

At the preoperative visit, ultrawide field (UWF) fundus photographs, FAF Daytona device (Optos, Marlborough, MA, USA), OCT (Spectralis, Heidelberg, Germany), and Solix (Optovue, Freemont, CA, USA) also were performed. Fundus drawings also were created since some patients had important vitreous haze what could affect image quality (Fig. 1).

On postoperative day 1, the eyes underwent BCVA measurement, biomicroscopy, fundus photographs, OCT, UWF fundus photography, and FAF. All examinations were performed on days 7, 30, and 90; the same examinations performed on day 1 were repeated. On days 30 and 90, patients also underwent UWF FA using the Optos California device. Two retina specialists (CAMN and MM) reviewed the images and performed the analysis; a third reviewer (MF) provided adjudication when necessary. Electroretinography (ERG) was not performed in this study due to the known limitations in eyes filled with silicone oil (SO), which attenuates light transmission and distorts electrophysiological responses [24, 25]. Future comparative studies including ERG, preferably with a control group using SO without melphalan, may provide valuable insight.

### Surgical procedures

We performed valved 23-gauge 4-port PPVs using the 10,000 cuts/min probe and a chandelier light pipe (Alcon, Fort Worth, TX, USA). A bimanual surgery using chromovitrectomy was performed to better identify vitreous in the periphery, hyaloid, and PVR; phacoemulsification and intraocular lens (IOL) aspheric implantation (Aspheric WF, AcrySof, Alcon) were performed for vitreous base shaving followed by scleral buckle number 42 and PPV for PVR removal with forceps in the six study eyes. IOL power calculation was based on immersion biometry using the OcuScan (Alcon), and corneal astigmatism was by spherical equivalent. The final IOL power was decreased by -0.50 diopter due to the myopic effect of the scleral buckle.

Following vitrectomy and fluid-air exchange and laser photocoagulation at the break sites, an intravitreal injection of 5 µg/0.1 cc of melphalan was followed by a normal-density 5,000-cs SO injection (Oxane, Bausch & Lomb, Bridgewater, NJ, USA). The same surgeon (MM) performed the standardized procedure in all eyes (Supplemental Video 1).

### Melphalan pharmaceutical Preparation

**Fig. 2:**
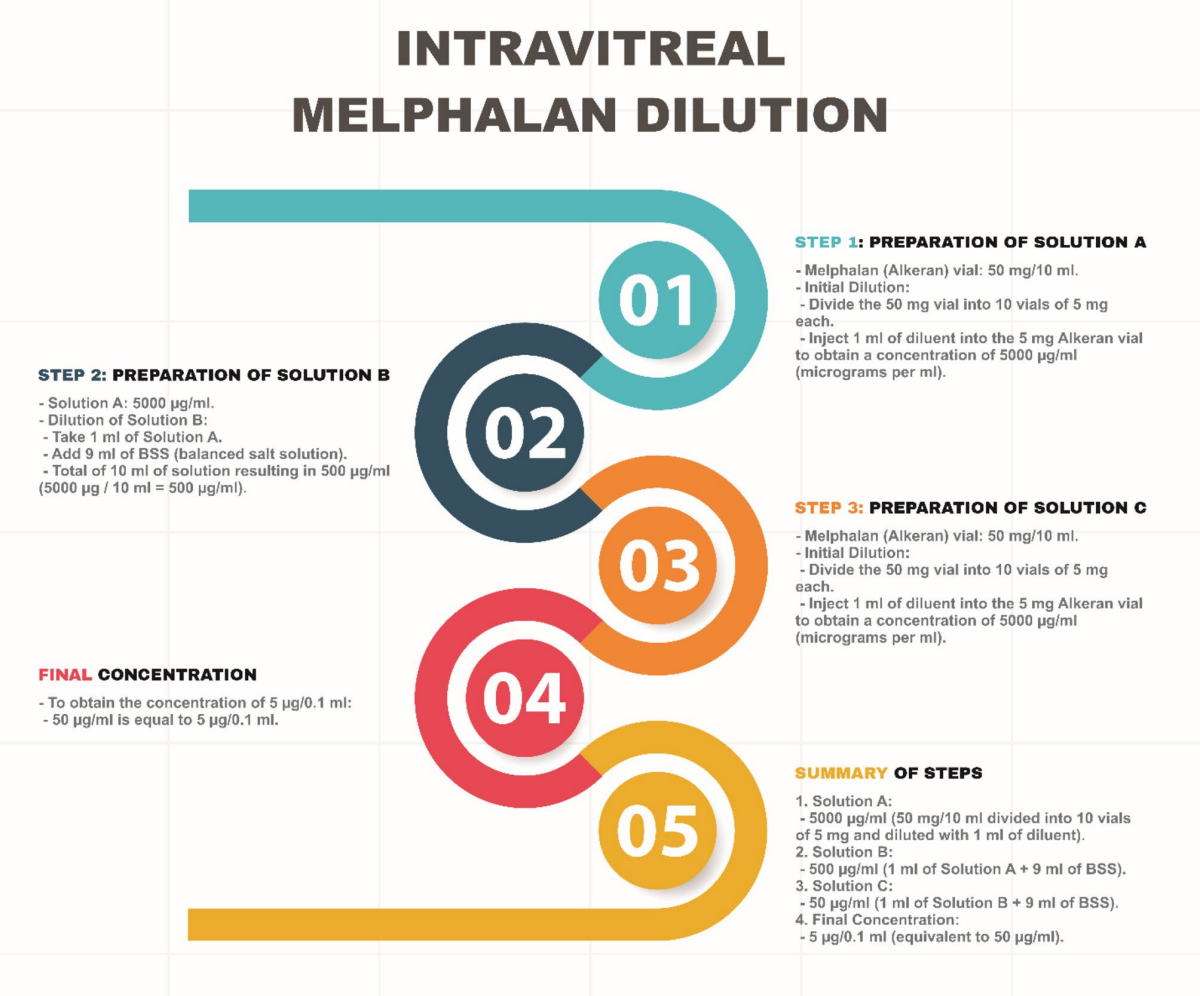
Schematic step-by-step representation of melphalan dilution

Melphalan is a highly unstable molecule and must be used 90 min after drug dilution. One vial of the lyophilized commercial product, Alkeran 50 mg (Aspen Pharma, Durban, South Africa) was weighed on a digital precision scale and aliquoted in 10 equal flasks of 5 mg of the lyophilized drug (Eyepharma Pharmaceutical Industry, Sao Paulo, Brazil) (Fig. 2). During PPV, just after the fluid air exchange, the same surgeon (MM) performed the dilution.

One vial was diluted gently in 1 ml of distilled water using a 1-ml BD Syringe (Becton Dickenson, Woburn, MA), resulting in 5 mg/ml or 5,000 ug/ml; 0.1 ml of the solution (500 ug) was diluted gently in 9.9 ml of balanced salt solution using a 10-ml BD syringe (Becton Dickenson), resulting in a 50 ug/ml solution. Finally, another 1-ml BD syringe was used to aspirate 0.1 ml of the solution resulting in 5 ug/0.1 ml (Fig. 2). Subsequently, the 0.1 ml (5 ug) of melphalan (osmolarity, 270 mOsm) was injected under air over the optic disc and fovea using a BD needle over the posterior pole under infusion air pressure of 10 mmHg, resulting in drops of melphalan at the posterior pole. Care was taken to inject slowly to avoid mechanical damage to the posterior pole structures and optic disc (Supplemental Video 1).

Finally, 5,000 cs of normal-density SO (Oxane 5000) was injected into the eye. Scleral sutures were placed at the 23-gauge trocar sites followed by second inverted sutures at the conjunctiva and Tenon’s capsule using vicryl 8.0 to minimize foreign-body sensation and buckle extrusion (Supplemental Video 1).

### Statistical analysis

The study data were compiled with descriptive statistics. Absolute and relative frequencies were calculated for categorical variables, and summary measures (mean, median, minimum, maximum and standard deviation) were determined for numerical variables. The mean BCVA values over time were assessed by analysis of variance. The normality of the data distribution was verified by the Kolmogorov-Smirnov test. A significance level of 5% was used for all statistical tests. The analyses were performed using the statistical software SPSS 20.0 (IBM Corp. Released 2011. IBM SPSS Statistics for Windows, Version 20.0, Armonk, NY, USA) and STATA 17 (StataCorp. 2021, Stata Statistical Software: Release 17, College Station, TX, USA).

For statistical analyses, the BCVA was expressed logMAR units. Non-parametric tests were used to compare the BCVAs; the non-parametric Friedman test was used because precision quantification of the BCVA was limited for some patients with hand motions and count fingers vision [21]. If differences in the BCVA levels were observed, Dunn-Bonferroni multiple comparisons were used to identify the times with different BCVA levels, maintaining the overall significance level. The analysis was performed using the SPSS 20.0 statistical software and data were shown on waterflow plots.

## Results

Six patients (5 men; median age, 64.5 years ± 12.2) were enrolled in the study. Two were Caucasian (33.33%) and four African-American (66.67%). The follow-up was 90 days.

**Fig. 3:**
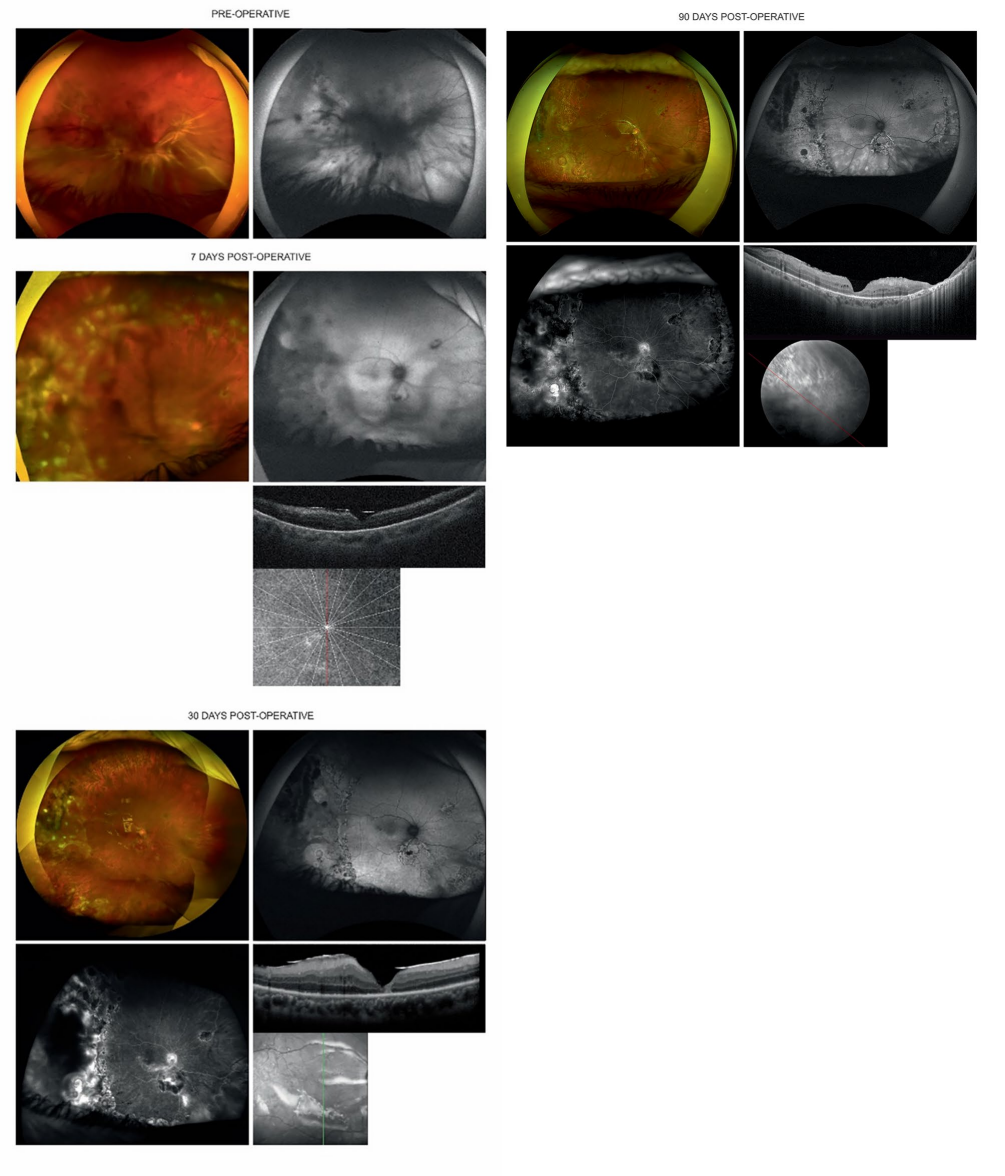
Images from patient 1 at various follow-up evaluations. Preoperatively: A, An UWF color fundus image shows vitreous opacities, macula off and inferior RD with a star pattern PVR CP6. B, An UWF FAF image shows a RD. Day 7 postoperatively, A, An UWF color fundus image shows vitreous opacities and a reattached retina. Laser scars are seen in the periphery. B, An UWF FAF image shows a reattached retina. C, A vertical B-scan SD-OCT image shows a reattached macula with absence of the outer retina. Day 30 postoperatively, A, An UWF color fundus image shows complete retinal reattachment. Laser scars are seen in the periphery. There are no signs of PVR recurrence. B, An UWF FAF image shows a reattached retina. Hypo-autofluorescent dots are present because of laser scars in the periphery and around the retinotomy beneath the optic disc. C, An UWF FA image shows leakage in the inferior part of the posterior pole. D, A vertical B-scan SD-OCT image shows foveal thinning. Small paracentral intraretinal cysts are seen at the level of the inner nuclear layer. Day 90 postoperatively, A, An UWF color fundus image shows a re-detachment of the inferior retina due to a posterior retinal tear (green arrow). Laser scars are seen in the periphery. There are no signs of PVR recurrence. B, An UWF FAF image shows re-detachment of the inferior retina. The retinal tear is hyper-autofluorescent (green arrow). C, An UWF FA image shows less leakage in the inferior posterior pole. D, An oblique B-scan SD-OCT image shows stability of both the foveal thinning and the paracentral intraretinal cysts

All patients had baseline BCVAs worse than 1.8 logMAR in the eye with the RRD. Only one patient had a previous history of cataract surgery. Fundus examination showed tobacco dust in the vitreous and macula-off RRD in all cases. Table 1 shows the patient characteristics including the PVR severity and extent and the presence/location of retinal tears (Table 1; Fig. 3).

**Table 1:** Comparison of preoperative and postoperative characteristics for each patient

Characteristic	Patient 1	Patient 2	Patient 3	Patient 4	Patient 5	Patient 6
Ethnicity/age/sex	AA/68/F	AA/79/M	CA/69/M	CA/47/M	AA/61/M	AA/50/M
BCVA (Pre) ETDRS (logMAR)	20/4000 (2.3)	20/4000 (2.3)	20/2000 (2.0)	20/1600 (1.9)	20/1400 (1.85)	20/4000 (2.3)
BCVA (PO90) ETDRS (logMAR)	20/800 (1.6)	20/100 (0.69)	20/50 (0.39)	20/200 (1.0)	20/100 (0.69)	20/160 (0.69)
Eyelids (Pre)	WNL	WNL	WNL	WNL	WNL	WNL
Eyelids (PO90)	WNL	WNL	WNL	WNL	WNL	WNL
Conjunctiva/sclera (Pre)	WNL	WNL	WNL	WNL	WNL	WNL
Conjunctiva/sclera (90PO)	WNL	WNL	WNL	WNL	WNL	WNL
Cornea (Pre)	WNL	WNL	WNL	WNL	WNL	WNL
Cornea (PO90)	WNL	WNL	WNL	WNL	WNL	WNL
Anterior chamber (Pre)	WNL	WNL	WNL	WNL	WNL	WNL
Anterior chamber (PO90)	WNL	WNL	WNL	Silicone oil bubble	WNL	WNL
Iris (Pre)	WNL	WNL	WNL	WNL	WNL	WNL
Iris (PO90)	WNL	WNL	WNL	WNL	WNL	WNL
Lens (Pre)	IOL	Phakic	Phakic	Phakic	Phakic	Phakic
Lens (PO90)	IOL	IOL	IOL	IOL	IOL	IOL
Vitreous (Pre)	Haze/RPE cells	Haze/RPE cells	Haze/RPE cells	Haze/RPE cells	Haze/RPE cells	Haze/RPE cells
Vitreous (PO90)	Silicone oil	Silicone oil	Silicone oil	Silicone oil	Silicone oil	Silicone oil
Optic disc (Pre)	POD, 0.4 c/d	POD, 0.4 c/d	POD, 0.3 c/d	POD, 0.3 c/d	POD, 0.3 c/d	POD, 0.3 c/d
Optic disc (PO90)	Stable	Stable	Stable	Stable	Stable	Stable
Macula (Pre)	Off	Off	Off	Off	Off	Off
Macula (PO90)	Macular sheen attenuation	WNL	WNL	WNL	Subretinal fibrosis	WNL
Peripheral retina (Pre)	Tear 10 o’clock, PVR CP6	Tear 3 o’clock, PVR CP3	Tear 7 o’clock, PVR CP3, temporal retinal cyst	Tear 2 o’clock, PVR CP2	Tears 3, 6, 9 o’clock, PVR CP6	Tears 5, 6, 8 o’clock, PVR CP2
Peripheral retina (PO90)	Laser blocking retinotomies tear and 360^o^	Laser blocking retinotomies tear and 360^o^	Laser blocking tear and 360^o^	Laser blocking retinotomy, tear, and 360^o^	Laser blocking retinotomy, tear and 360^o^	Laser blocking retinotomy, tear and 360^o^
UWF FA (PO90)	Peripheral leakage	Peripheral leakage and staining	Peripheral leakage	Posterior pole leakage	Peripheral leakage	--
OCT (PO90)	Intraretinal cysts. Retinal thinning	Intraretinal cyst. Outer retina recuperation. Attenuated foveal depression	Outer retina recovery.	Intraretinal cysts. Outer retina recovery. ERM	Intraretinal cysts. Outer retina discontinuation	Outer retina recovery

*Pre* preoperative, *PO* postoperative, *AA* Afro-American, *CA* Caucasian, *F* female, *M* male, *BCVA* best-corrected visual acuity, *IOP* intraocular pressure, *WNL* within normal limits, *IOL* intraocular lens, RPE retinal pigment epithelium, *POD* pink optic disc, *C/D* cup-to-disc ratio, *ERM* epiretinal membrane

All patients underwent surgery according to the described protocol. At the end of the surgery, the retinas were reattached in all eyes (Figs. 1 and 3).

On day 1 postoperatively, all six patients reported mild pain. On biomicroscopy, mild conjunctival injection, mild-to-moderate corneal edema, and a mild anterior chamber reaction (ACR) were seen in all eyes. A SO bubble in the anterior chamber was seen in one patient. The IOP was within the normal limits in all eyes.

**Fig. 4:**
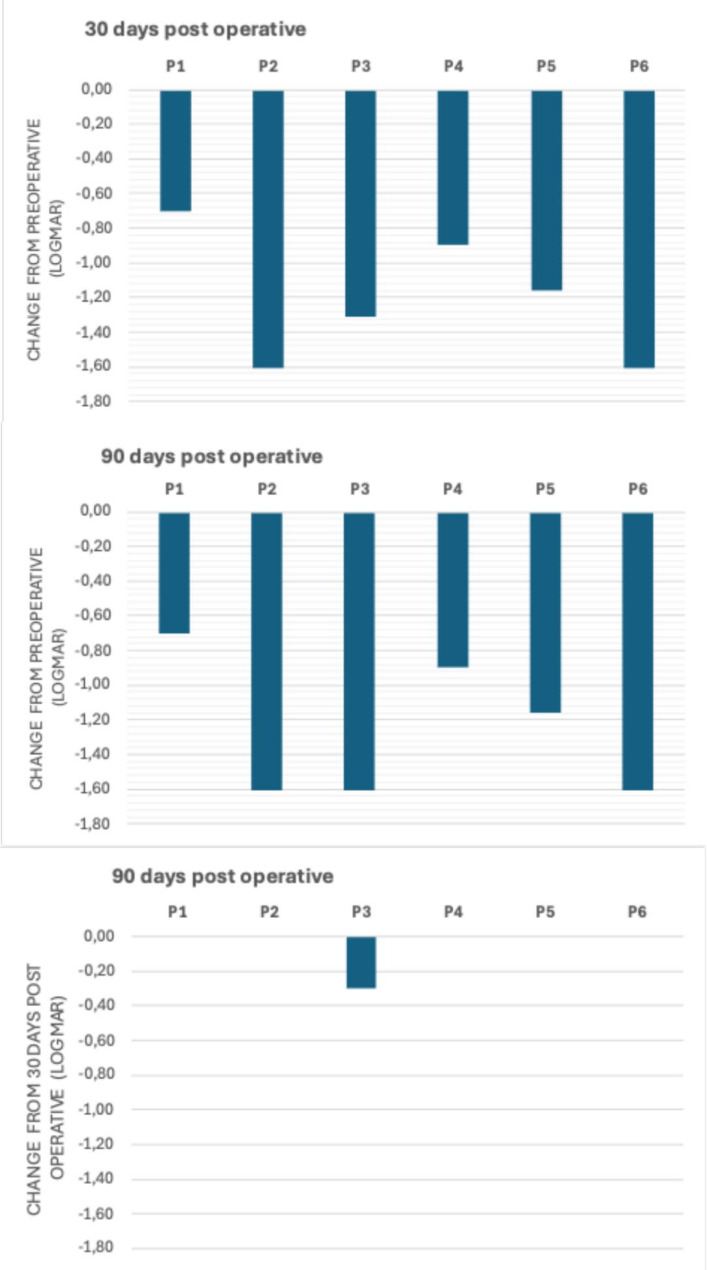
Waterfall plots show variation in the logMAR BCVA using the parametric Friedman and Dunn-Bonferroni tests

On postoperative day 7 (Fig. 3), the corneal edema improved in most eyes. The ACR was stable. The iris color did not change in any eye. Patient 4 with a SO bubble in the anterior chamber had an IOP increase to 40 mmHg, for which oral and topical hypotensive agents were prescribed. The posterior segment evaluation showed that the vitreous cavity in all patients was filled with SO, the retina was reattached, there were no signs of PVR, the optic disc color was normal, and no vascular abnormalities or pigment changes were seen. Patient 1 had macular sheen attenuation (Fig. 3), which was not attributed to melphalan. Figure 4 shows images obtained on postoperative day 7.

On day 30 postoperatively, biomicroscopy showed resolution of the conjunctival injection and ACR in most eyes. Only eyes 2 and 3 had a mild ACR. The IOP in eye 4 was controlled by topical and oral drugs. Eye 1 maintained the same macular characteristics observed on postoperative day 7 (Fig. 3). UWF FA showed leakage; OCT showed varying numbers of intraretinal cysts and outer retinal atrophy in all patients (Table 1; Fig. 3).

On postoperative day 90, the BCVA was stable and improved in all eyes. No eyes had conjunctival injection or an ACR. OCT and UWF FA did not show new findings compared to postoperative day 30. At the final follow-up visit, no signs of pigmentary changes, vasculitis, or optic neuropathy were detected in any patient, supporting the absence of clinical retinal toxicity from melphalan at this dosage. (Figures 1 and 3; Table 1).

Figure 4 (parametric test) shows the BCVA improvements according to the progressive time lines (*P* < 0.001). The initial mean BCVA (2.11 ± 0.22) was worse compared to days 30 (0.89 ± 0.37) and 90 postoperatively (0.84 ± 0.42), which were similar (*P* < 0.001).

A non-parametric test analyzed the different BCVA levels throughout the evaluations. The median preoperative BCVA level (2.15) was worse than on days 30 (0.69) and 90 (0.69) (*P* = 0.003), which were similar to each other.

## Discussion

PVR, a major complication in surgical retina, often leads to unsuccessful treatments and global atrophy in complex cases [12]. Despite advances, no gold standard treatment exists, and intraocular drugs have failed to control it [26, 27]. To our best knowledge, this is the first study to perform PPV and scleral buckle associated with intraocular melphalan as a therapeutic option for RRD with PVR (Medline search, July 9, 2024).

In six study patients, no intraocular inflammation or toxicity was directly linked to intraocular melphalan. Conjunctival injection and mild ACR were expected after scleral buckling and PPV, while peripheral leakage was attributed to prolonged intraocular surgery for PVR related to RRD, not melphalan. Postoperative OCT scans commonly showed outer retinal discontinuation, typical in long-term RD cases. Cystoid macular edema was observed in some patients, a common outcome of similar surgeries, such as cataract surgery, without melphalan [6, 10, 11, 28].

Melphalan, studied in animals and humans for various intraocular diseases, was chosen for retinoblastoma treatment due to its ability to inhibit colony formation [19]. It is ideal as a regional chemotherapeutic agent because of its short half-life, low tissue toxicity, and a linear dose-response relationship with cytotoxicity [29].

Buitrago et al. studied intravitreal melphalan in a rabbit model and reported its ability to achieve high vitreous levels, enhancing bioavailability and effectiveness for retinoblastoma treatment. The peak concentrations were 7.8 mg/dL in the vitreous, 0.024 mg/dL in the aqueous humor, and 9.8 mg/g in the retina, with half-lives of 1.0, 0.2, and 1.2 h, respectively. Melphalan remained active in the vitreous for 5 h and detectable for 12 h, with minimal systemic exposure, suggesting a low risk of systemic toxicity. This supports intravitreal melphalan as an effective localized treatment [30]. In our study, the intravitreal injection of melphalan was performed under air, prior to SO filling. While exact pharmacokinetics remain to be established, the injected drug likely settled at the posterior pole before being displaced by SO. We hypothesize that this method leads to a short-term localized exposure at higher concentrations over the macula, followed by dilution and dispersion within the SO-filled cavity. Pharmacokinetic studies are encouraged to quantify this exposure.

In 2014, Francis et al. found that intravitreal melphalan (30 µg weekly) caused no systemic toxicity but led to permanent retinal dysfunction with decreased electroretinography (ERG) responses [31]. In the current study, ERG was not performed due to limitations with SO-filled eyes [24, 25], but improvements in the BCVA, visual fields, OCT, FA, FAF, and fundus imaging showed no signs of melphalan toxicity. Although one eye had a minor tear, no signs of PVR recurrence were observed in any patient during the follow-up period, suggesting potential safety and efficacy of melphalan. A longer-term follow-up protocol, including SO removal, is currently ongoing and will be reported separately. These preliminary results highlight the promise of melphalan in PVR management and support the need for further prospective studies. The outer retinal thinning and intraretinal cysts observed in OCT imaging, as well as peripheral leakage on fluorescein angiography, are common findings in chronic RD cases and postoperative eyes. They are not specific for melphalan toxicity, and similar patterns are observed in eyes undergoing complex RD repair without adjuvant drug therapy. Additionally, the occurrence of cystoid macular edema in this cohort aligns with known postoperative complications of PPV and scleral buckle surgery. The incidence was not unusually high, and given the low melphalan dose used, drug-related causality is unlikely.

Suzuki et al. reviewed 264 eyes of 250 retinoblastoma patients treated with 1,067 intravitreal melphalan injections (16–24 µg) from 1990 to 2011 and found a low risk of adverse effects, with 68% achieving complete remission of vitreous seeds and 50% maintaining functional vision (0.5). Intravitreal melphalan was deemed safe and effective for treating vitreous seeds [32].

Shimoda et al. studied retinal changes in rabbits after intravitreal melphalan during PPV and found that a 5-µg dose caused no ERG or histologic changes. A 10-µg dose led to a 65–68% decrease in ERG a- and b-waves on day 3, with mild histologic damage, including photoreceptor defects. A 20-µg dose caused severe retinal degeneration, with nearly flat ERG waves by day 28. Based on these findings, the authors chose a 5-µg dose for the current study [19]. Despite the impossibility of performing ERG in the current eyes because of the SO tamponade, all eyes had BCVA improvements, demonstrating no severe functional loss due to the use of 5 µg/0.1 ml melphalan. Furthermore, Shimoda et al. demonstrated that 5 µg of intravitreal melphalan caused no ERG or histological damage in rabbit eyes, supporting the safety of this dose. Given that ERG signals are significantly dampened in SO-filled eyes, we deemed it unreliable for toxicity screening in this setting. However, future studies with control groups using SO without melphalan could better isolate the functional impact of the drug.

In a study of 12 cases treated with intravitreal melphalan, doses of 8 to 10 µg caused minor complications like preretinal hemorrhages and retinal vasculitis. However, at 50 µg, serious complications such as cataracts, vitreous hemorrhage, subretinal hemorrhage, severe hypotony, and enucleation occurred, indicating toxicity at higher doses. [33]. In the current study, 3 months after the use of melphalan during PPV, there were no signs of preretinal hemorrhage, vasculitis, RPE changes, or neuritis.

Francis et al. evaluated 600 intravitreal melphalan injections (25–30 µg) for retinoblastoma, some combined with topotecan and found that eyes with greater iris and fundus pigmentation may absorb more melphalan, leading to increased RPE, retina, and choroid toxicity. The study concluded that each injection resulted in a decrease of about 5 mV in the ERG response [34]. Four of the current six patients were African-Americans and none showed RPE pigmentation or other sign of toxicity within 3 months of follow-up. We hypothesized that this absence of toxicity could have resulted from the lower melphalan dose than in the study of Francis et al.

In 2016, Francis et al. also analyzed 76 patients treated with intraocular melphalan (doses, 20–30 µg). Five cases had anterior segment complications (traumatic cataract, iris depigmentation, and focal scleromalacia) [34]. No current patients had these side effects, which we hypothesized resulted from the 5 µg/0.1 ml dose of melphalan.

In summary, no intraocular inflammation was directly attributed to melphalan, and no recurrence of PVR was observed in these complex cases. All patients demonstrated improvements in BCVA. This pilot study was specifically designed to assess short-term safety and feasibility, which justified the limited sample size and the absence of a control group. These constraints were intentional, aiming to minimize risk in a high-risk population and to generate preliminary safety data prior to a larger trial. The short follow-up period and the lack of ERG testing should also be recognized as limitations. Although the inclusion of a control group would have strengthened the study, enrolling untreated patients in this context raised ethical concerns. A prospective controlled study is currently under development. Despite one eye developing a minor tear and localized subretinal fluid, the absence of toxicity or PVR recurrence over 90 days reinforces the potential safety of melphalan, though further investigation with a more robust study design is warranted.

The continuity of the pilot study, including SO removal and a clinical trial are now planned.

## Conclusions

The study showed promising short-term safety for intravitreal 5 µg/0.05 ml melphalan combined with PPV and SO tamponade in treating RRD with PVR. No toxicity was observed from the melphalan injection at the posterior pole. The findings suggest that melphalan could be an alternative to prevent PVR recurrence, although further studies with control groups, more eyes, and longer follow-up are recommended.

## Electronic supplementary material

Below is the link to the electronic supplementary material.

Supplementary Material 1: **Supplementary video**: Surgical steps: (1) Scleral buckle number 42; (2) 23 g PPV; (3) PVR removal; 4)PFO use, 5) removal of the vitreous base woth scleral identation and chandelier accessory lighting, 6) retinotomies, 7) air fluid exchange, 8) laser barrier, 9) melphalan injection over the posterior pole, 10) silicon oil injection, 11) scleral and conjunctival sutures

## Data Availability

The databases generated and analyzed during the current study are available from the corresponding author upon reasonable request.

## References

[1] Greven CM, Sanders RJ, Brown GC, et al. Pseudophakic retinal detachments. Anatomic and visual results. Ophthalmol Feb. 1992;99(2):257–62. 10.1016/s0161-6420(92)31983-9. 10.1016/s0161-6420(92)31983-9 1553218

[2] Girard P, Mimoun G, Karpouzas I, Montefiore G. Clinical risk factors for proliferative vitreoretinopathy after retinal detachment surgery. Retina. 1994;14(5):417–24. 10.1097/00006982-199414050-00005. 7899716 10.1097/00006982-199414050-00005

[3] Gartry DS, Chignell AH, Franks WA, Wong D. Pars plana vitrectomy for the treatment of rhegmatogenous retinal detachment uncomplicated by advanced proliferative vitreoretinopathy. Br J Ophthalmol Apr. 1993;77(4):199–203. 10.1136/bjo.77.4.199. 10.1136/bjo.77.4.199 PMC504480 8494853

[4] Bonnet M, Fleury J, Guenoun S, Yaniali A, Dumas C, Hajjar C. Cryopexy in primary rhegmatogenous retinal detachment: a risk factor for postoperative proliferative vitreoretinopathy? Graefes Arch Clin Exp Ophthalmol Dec. 1996;234(12):739–43. 10.1007/BF00189354. 10.1007/BF00189354 8986445

[5] Ge JY, Teo ZL, Chee ML, et al. International incidence and Temporal trends for rhegmatogenous retinal detachment: A systematic review and meta-analysis. Surv Ophthalmol May-Jun. 2024;69(3):330–6. 10.1016/j.survophthal.2023.11.005. 10.1016/j.survophthal.2023.11.005 38000699

[6] Charteris DG, Sethi CS, Lewis GP, Fisher SK. Proliferative vitreoretinopathy-developments in adjunctive treatment and retinal pathology. Eye (Lond) Jul. 2002;16(4):369–74. 10.1038/sj.eye.6700194. 10.1038/sj.eye.6700194 12101443

[7] Speicher MA, Fu AD, Martin JP, von Fricken MA. Primary vitrectomy alone for repair of retinal detachments following cataract surgery. Retina. 2000;20(5):459–64. 10.1097/00006982-200009000-00005. 11039419 10.1097/00006982-200009000-00005

[8] Duquesne N, Bonnet M, Adeleine P. Preoperative vitreous hemorrhage associated with rhegmatogenous retinal detachment: a risk factor for postoperative proliferative vitreoretinopathy? Graefes Arch Clin Exp Ophthalmol Nov. 1996;234(11):677–82. 10.1007/BF00292353. 10.1007/BF00292353 8950587

[9] Kwon OW, Song JH, Roh MI. Retinal detachment and proliferative vitreoretinopathy. Dev Ophthalmol. 2016;55:154–62. 10.1159/000438972. 26501375 10.1159/000438972

[10] Sadaka A, Giuliari GP. Proliferative vitreoretinopathy: current and emerging treatments. Clin Ophthalmol. 2012;6:1325–33. 10.2147/OPTH.S27896. 22942638 10.2147/OPTH.S27896 PMC3429288

[11] Pastor JC, de la Rua ER, Martin F. Proliferative vitreoretinopathy: risk factors and pathobiology. Prog Retin Eye Res Jan. 2002;21(1):127–44. 10.1016/s1350-9462(01)00023-4. 10.1016/s1350-9462(01)00023-4 11906814

[12] Pastor JC. Proliferative vitreoretinopathy: an overview. *Surv ophthalmol*. Jul-Aug. 1998;43(1):3–18. 10.1016/s0039-6257(98)00023-x. 10.1016/s0039-6257(98)00023-x 9716190

[13] Bringmann A, Wiedemann P. Involvement of Muller glial cells in epiretinal membrane formation. Graefes Arch Clin Exp Ophthalmol Jul. 2009;247(7):865–83. 10.1007/s00417-009-1082-x. 10.1007/s00417-009-1082-x 19415318

[14] Pastor JC, Rojas J, Pastor-Idoate S, Di Lauro S, Gonzalez-Buendia L, Delgado-Tirado S. Proliferative vitreoretinopathy: A new concept of disease pathogenesis and practical consequences. Prog Retin Eye Res Mar. 2016;51:125–55. 10.1016/j.preteyeres.2015.07.005. 10.1016/j.preteyeres.2015.07.005 26209346

[15] Poczta A, Rogalska A, Marczak A. Treatment of multiple myeloma and the role of Melphalan in the era of modern Therapies-Current research and clinical approaches. J Clin Med Apr. 2021;23(9). 10.3390/jcm10091841. 10.3390/jcm10091841 PMC8123041 33922721

[16] Conteduca V, Scarpi E, Farolfi A, et al. Melphalan as a promising treatment for BRCA-Related ovarian carcinoma. Front Oncol. 2021;11:716467. 10.3389/fonc.2021.716467. 34367999 10.3389/fonc.2021.716467 PMC8336462

[17] Jorge R, Coelho I, Viani G, et al. Melphalan intra-arterial chemotherapy for choroidal melanoma chemoreduction. Int J Retina Vitreous Aug. 2022;17(1):55. 10.1186/s40942-022-00404-1. 10.1186/s40942-022-00404-1 PMC9386917 35978407

[18] Dahi PB, Lin A, Scordo M, et al. Evaluation of Melphalan exposure in lymphoma patients undergoing BEAM and autologous hematopoietic cell transplantation. Transpl Cell Ther Aug. 2022;28(8):485. 10.1016/j.jtct.2022.05.003. e1-485 e6. 10.1016/j.jtct.2022.05.003 PMC9357179 35545213

[19] Shimoda Y, Hamano R, Ishihara K, et al. Effects of intraocular irrigation with Melphalan on rabbit retinas during vitrectomy. Graefes Arch Clin Exp Ophthalmol Apr. 2008;246(4):501–8. 10.1007/s00417-007-0685-3. 10.1007/s00417-007-0685-3 17934752

[20] Machemer R, Aaberg TM, Freeman HM, Irvine AR, Lean JS, Michels RM. An updated classification of retinal detachment with proliferative vitreoretinopathy. Am J Ophthalmol Aug. 1991;15(2):159–65. 10.1016/s0002-9394(14)76695-4. 10.1016/s0002-9394(14)76695-4 1867299

[21] Schulze-Bonsel K, Feltgen N, Burau H, Hansen L, Bach M. Visual acuities hand motion and counting fingers can be quantified with the Freiburg visual acuity test. Invest Ophthalmol Vis Sci Mar. 2006;47(3):1236–40. 10.1167/iovs.05-0981. 10.1167/iovs.05-0981 16505064

[22] Moussa G, Bassilious K, Mathews N. A novel excel sheet conversion tool from Snellen fraction to LogMAR including ‘counting fingers’, ‘hand movement’, ‘light perception’ and ‘no light perception’ and focused review of literature of low visual acuity reference values. Acta Ophthalmol Sep. 2021;99(6):e963–5. 10.1111/aos.14659. 10.1111/aos.14659 33326177

[23] Patel H, Congdon N, Strauss G, Lansingh C. A need for standardization in visual acuity measurement. Arq Bras Oftalmol Sep-Oct. 2017;80(5):332–7. 10.5935/0004-2749.20170082. 10.5935/0004-2749.20170082 29160549

[24] Azarmina M, Soheilian M, Azarmina H, Hosseini B. Electroretinogram changes following silicone oil removal. J Ophthalmic Vis Res Apr. 2011;6(2):109–13. PMC3306082 22454719

[25] Ozaki K, Yoshikawa Y, Ishikawa S, et al. Electroretinograms recorded with skin electrodes in silicone oil-filled eyes. PLoS ONE. 2019;14(5):e0216823. 10.1371/journal.pone.0216823. 31150414 10.1371/journal.pone.0216823 PMC6544342

[26] Wickham L, Bunce C, Wong D, McGurn D, Charteris DG. Randomized controlled trial of combined 5-Fluorouracil and low-molecular-weight heparin in the management of unselected rhegmatogenous retinal detachments undergoing primary vitrectomy. Ophthalmol Apr. 2007;114(4):698–704. 10.1016/j.ophtha.2006.08.042. 10.1016/j.ophtha.2006.08.042 17398320

[27] Asaria RH, Kon CH, Bunce C, et al. Adjuvant 5-fluorouracil and heparin prevents proliferative vitreoretinopathy: results from a randomized, double-blind, controlled clinical trial. Ophthalmol Jul. 2001;108(7):1179–83. 10.1016/s0161-6420(01)00589-9. 10.1016/s0161-6420(01)00589-9 11425671

[28] Pennock S, Rheaume MA, Mukai S, Kazlauskas A. A novel strategy to develop therapeutic approaches to prevent proliferative vitreoretinopathy. Am J Pathol Dec. 2011;179(6):2931–40. 10.1016/j.ajpath.2011.08.043. 10.1016/j.ajpath.2011.08.043 PMC3260857 22035642

[29] Defty CL, Marsden JR. Melphalan in regional chemotherapy for locally recurrent metastatic melanoma. Curr Top Med Chem. 2012;12(1):53–60. 10.2174/156802612798919187. 22196271 10.2174/156802612798919187

[30] Buitrago E, Winter U, Williams G, Asprea M, Chantada G, Schaiquevich P. Pharmacokinetics of Melphalan after intravitreal injection in a rabbit model. J Ocul Pharmacol Ther May. 2016;32(4):230–5. 10.1089/jop.2015.0088. 10.1089/jop.2015.0088 26785130

[31] Francis JH, Schaiquevich P, Buitrago E, et al. Local and systemic toxicity of intravitreal Melphalan for vitreous seeding in retinoblastoma: a preclinical and clinical study. Ophthalmol Sep. 2014;121(9):1810–7. 10.1016/j.ophtha.2014.03.028. 10.1016/j.ophtha.2014.03.028 24819859

[32] Suzuki S, Aihara Y, Fujiwara M, Sano S, Kaneko A. Intravitreal injection of Melphalan for intraocular retinoblastoma. Jpn J Ophthalmol May. 2015;59(3):164–72. 10.1007/s10384-015-0378-0. 10.1007/s10384-015-0378-0 25808017

[33] Ghassemi F, Shields CL. Intravitreal Melphalan for refractory or recurrent vitreous seeding from retinoblastoma. Arch Ophthalmol Oct. 2012;130(10):1268–71. 10.1001/archophthalmol.2012.1983. 10.1001/archophthalmol.2012.1983 23044940

[34] Francis JH, Brodie SE, Marr B, Zabor EC, Mondesire-Crump I, Abramson DH. Efficacy and toxicity of intravitreous chemotherapy for retinoblastoma: Four-Year experience. Ophthalmol Apr. 2017;124(4):488–95. 10.1016/j.ophtha.2016.12.015. 10.1016/j.ophtha.2016.12.015 PMC5441308 28089679

